# Web-Based Problem-solving Training With and Without Peer Support in Veterans With Unmet Mental Health Needs: Pilot Study of Feasibility, User Acceptability, and Participant Engagement

**DOI:** 10.2196/29559

**Published:** 2022-01-13

**Authors:** Kyle Possemato, Justina Wu, Carolyn Greene, Rex MacQueen, Daniel Blonigen, Michael Wade, Jason Owen, Terence Keane, Deborah Brief, Steven Lindley, Annabel Prins, Margaret-Anne Mackintosh, Eve Carlson

**Affiliations:** 1 Veterans Affairs Center for Integrated Healthcare Syracuse, NY United States; 2 National Center for Post Traumatic Stress Disorder Veterans Affairs Palo Alto Health Care System Palo Alto, CA United States; 3 Veterans Affairs Office of Mental Health Services and Suicide Prevention Washington, DC United States; 4 Veterans Affairs Palo Alto Healthcare System Palo Alto, CA United States; 5 National Center for Post Traumatic Stress Disorder Veterans Affairs Boston Healthcare System Boston, MA United States

**Keywords:** problem-solving training, mHealth, peer specialists, veterans

## Abstract

**Background:**

eHealth tools have the potential to meet the mental health needs of individuals who experience barriers to accessing in-person treatment. However, most users have less than optimal engagement with eHealth tools. Coaching from peer specialists may increase their engagement with eHealth.

**Objective:**

This pilot study aims to test the feasibility and acceptability of a novel, completely automated web-based system to recruit, screen, enroll, assess, randomize, and then deliver an intervention to a national sample of military veterans with unmet mental health needs; investigate whether phone-based peer support increases the use of web-based problem-solving training compared with self-directed use; and generate hypotheses about potential mechanisms of action for problem-solving and peer support for future full-scale research.

**Methods:**

Veterans (N=81) with unmet mental health needs were recruited via social media advertising and enrolled and randomized to the self-directed use of a web-based problem-solving training called Moving Forward (28/81, 35%), peer-supported Moving Forward (27/81, 33%), or waitlist control (26/81, 32%). The objective use of Moving Forward was measured with the number of log-ins. Participants completed pre- and poststudy measures of mental health symptoms and problem-solving confidence. Satisfaction was also assessed post treatment.

**Results:**

Automated recruitment, enrollment, and initial assessment methods were feasible and resulted in a diverse sample of veterans with unmet mental health needs from 38 states. Automated follow-up methods resulted in 46% (37/81) of participants completing follow-up assessments. Peer support was delivered with high fidelity and was associated with favorable participant satisfaction. Participants randomized to receive peer support had significantly more Moving Forward log-ins than those of self-directed Moving Forward participants, and those who received peer support had a greater decrease in depression. Problem-solving confidence was associated with greater Moving Forward use and improvements in mental health symptoms among participants both with and without peer support.

**Conclusions:**

Enrolling and assessing individuals in eHealth studies without human contact is feasible; however, different methods or designs are necessary to achieve acceptable participant engagement and follow-up rates. Peer support shows potential for increasing engagement in web-based interventions and reducing symptoms. Future research should investigate when and for whom peer support for eHealth is helpful. Problem-solving confidence should be further investigated as a mechanism of action for web-based problem-solving training.

**Trial Registration:**

ClinicalTrials.gov NCT03555435; http://clinicaltrials.gov/ct2/show/NCT03555435

## Introduction

### Background

Many Americans have mental health needs that go unmet. A national survey found that 11.8 million US adults report unmet mental health needs, with about half of these respondents reporting that they received some, but not enough, mental health services to meet their needs, and about half reporting that they received no mental health services [1]. Even in health care systems that screen for mental health problems and offer treatment, such as the Veterans Health Administration (VHA) and the Department of Defense (DoD), there are substantial numbers of individuals with unmet mental health needs [2,3]. In a study of veterans who screened positive for depression and posttraumatic stress disorder (PTSD) and were offered mental health care, less than half received any mental health treatment [4]. Barriers to treatment engagement include concerns with stigma, a desire to be self-reliant, a lack of appeal of face-to-face therapy, and the inconvenience of traveling to an appointment during the day [5,6]. More recently, health and safety concerns surrounding the COVID-19 pandemic emerged as new barriers to traditional psychotherapy [7].

Novel approaches are needed to engage individuals with unmet needs in mental health care. Electronic or *eHealth* tools are increasingly being used, given their ability to reach many users at relatively low costs, and growing evidence shows that many individuals prefer to use eHealth as part of their health care [8,9]. eHealth could dramatically diminish access problems related to travel distance and time, wait times for appointments, financial burden, stigma, and desire for self-reliance [10].

Websites and mobile apps that help individuals manage their mental health concerns are now widely available, and multiple meta-analyses have described their benefits for users [11,12]. One such program is Moving Forward (MF), a free educational and life coaching program that is a web-based adaptation of problem-solving therapy. MF was built by VHA in partnership with the DoD as part of a coordinated public health initiative to help veterans and service members who have difficulties with mental health. It was first made publicly available in 2011, and a program evaluation found that 750 veterans accessed it in 2020. Problem-solving therapy is an evidence-based, transdiagnostic cognitive behavioral treatment that has been successfully adapted to multiple contexts, including problem-solving training for primary care patients [13]. The intervention has a robust evidence base for a variety of disorders and is among the recommended treatments for depression and suicide prevention in the VHA/DoD Clinical Practice Guidelines. Problem-solving therapy is particularly well-suited to individuals who see their distress as a result of life problems rather than a mental health problem and want to solve their problems themselves.

The benefits gained from eHealth interventions tend to vary by how much an intervention is used, how well users apply the learned strategies to their daily lives, and users’ confidence in their ability to manage their mental health problems, known as mental health self-efficacy [14,15]. High rates of attrition in eHealth are ubiquitous, leaving many users to experience no benefits from the intervention [16]. On the basis of these findings, the supportive accountability model was developed to guide how human coaching can increase adherence to eHealth interventions. This model predicts higher engagement in eHealth when the user feels accountable to a coach who is seen as trustworthy and benevolent and having relevant expertise [17].

The unique characteristics of peer specialists may allow them to be particularly effective as eHealth coaches. Peer specialists are individuals who have lived experience with medical or mental health conditions, have benefitted from treatment, can model healthy behaviors, and can provide emotional support. A recent systematic review of 30 studies found that peer support (PS) of eHealth interventions has strong potential for clinical effectiveness; however, more research was recommended to investigate whether and how PS affects user engagement in eHealth and what mechanisms are related to better health among users [18]. The VHA has made a large investment in its peer workforce. Veteran peers now assist other veterans in coping with medical and mental health conditions, including providing support for the VHA’s suite of mental health apps and websites [19]. Research is needed to understand the best ways to deliver PS of eHealth tools and how processes to connect veterans with eHealth and peers can be automated.

### Objectives

This study is designed to test novel methods of delivering care and generate hypotheses for future research on eHealth delivery. The first aim of this study is to test the feasibility and user-perceived acceptability of the research and intervention methods. Specifically, we test the feasibility of an automated electronic system for recruitment, enrollment, intervention delivery, and data collection in a national sample of veterans recruited via social media. We test user-perceived acceptability via participant engagement in MF and peer sessions, as well as user satisfaction. The second aim is to examine engagement in the web-based MF course for those with and without PS. We hypothesize that the use of MF would be greater for the PS condition than for the self-directed (SD) condition. The third aim is to conduct an exploratory examination of changes in problem-solving skills and problem-solving confidence as possible mechanisms of action to increase engagement in MF or reduce mental health symptoms. The fourth aim is to conduct an exploratory examination of the association of PS sessions with changes in mental health symptoms. This study adds to the early research on how PS can enhance engagement in eHealth and makes unique contributions by investigating a fully automated system for research and interventions and exploring potential mechanisms of action.

## Methods

This study was found to meet all human subjects, data security, and privacy requirements for research approval by the local institutional research boards where the study investigators were located. The ClinicalTrials.gov identifier is NCT03555435.

### Participants and Procedures

**Figure 1:**
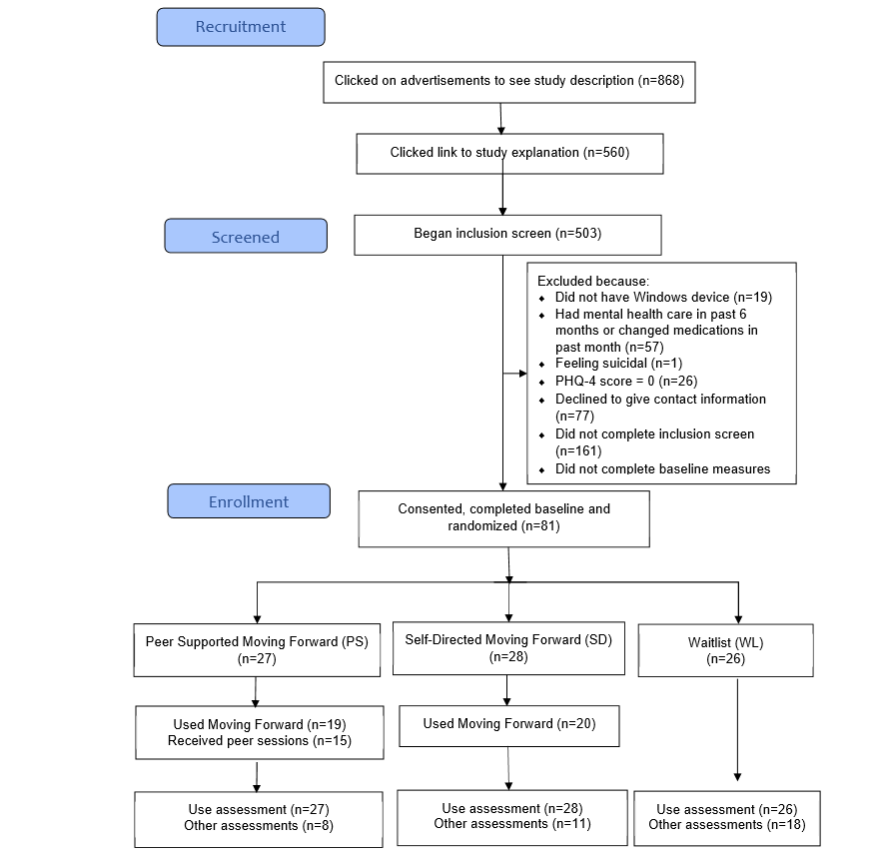
CONSORT (Consolidated Standards of Reporting Trials) flowchart. PHQ-4: Patient Health Questionnaire-4.

Veterans with unmet mental health needs were recruited for this study via Facebook advertisements. Figure 1 shows the flow of participants from having a Facebook advertisement show up on their Facebook feed to the collection of follow-up assessments. For this study, a veteran was determined to have unmet mental health needs if they endorsed mental health symptoms on assessment measures and denied engagement in current mental health treatment. To be included in the study, participants needed to (1) self-identify as veterans, (2) endorse any depression or anxiety item on the 4-item Patient Health Questionnaire [20] as occurring for several days or more in the past week, (3) have a phone and a computer with internet access, (4) be willing to be randomized to 1 of 3 conditions, and (5) work with a peer. Veterans were excluded if they had (1) an active suicide plan, (2) changes in psychoactive medications in the past month, or (3) were receiving mental health care at the time of enrollment. We confirmed veteran status using methods previously established by Kramer et al [21], which involve asking for details of service and contact information. Advertisements targeting veterans appeared in the Facebook feeds of users identified as having an interest in veterans. The advertisements were linked to a website that provided information about the study and allowed viewers to opt for a screening that was conducted via a web-based data collection tool, REDCap (Research Electronic Data Capture) [22,23]. The system recorded time stamps for when enrollments occurred to determine how often participants used the system outside of typical business hours within their home time zone.

Those who were eligible for the study completed baseline measures on the web, including assessment of sociodemographic characteristics, mental health symptoms, and problem-solving characteristics. Participants were randomized by the web-based platform to 1 of 3 conditions: SD MF, PS MF, and a waitlist (WL) control group. The WL group received no intervention and participated in both study assessments before being offered access to the MF course with or without PS. After their baseline assessment, SD and PS participants were directed to MF and asked to complete the 5 modules over the next 8 weeks. PS participants also received up to 5 phone sessions with a peer.

Course use data were collected using a commercial web-based learning platform. Collection was automated via programming, which initiated daily use reports to be sent from the learning platform to a REDCap file. To have use data identified with anonymous study IDs, the enrollment of each participant triggered a REDCap application programming interface to send the participant’s study ID number to a server programmed to set up a new user account in the web-based learning system. In this way, use data were automatically and anonymously associated with participant IDs. Programming was used to link the data management system to a web-based learning platform to study course use. The data management system also supported the delivery of peer services.

Symptoms and problem-solving measures were re-administered on the web at the end of the study (8 weeks post baseline), along with a satisfaction measure. Participants received 1 to 3 automated reminder emails over a 1- to 2-week period (spaced 3-5 days apart) with a link to complete follow-up measures. Participants who did not complete the assessment in the SD and WL conditions and some participants in the PS condition received at least one reminder phone call over a 2-week period (25/81, 31% were left voicemails, 27/81, 33% spoke directly to research staff, and 11/81,13% could not be reached). Peers reminded PS participants to complete the assessment at the final peer session (when this occurred). After the final follow-up assessment, participants were given a choice between ending study participation and agreeing to a qualitative phone interview. Participants were compensated with gift cards and were paid US $5 for answering baseline measures, US $10 for completing the end of study measures, and US $15 for completing a qualitative interview (US $30 maximum).

### Measures

To make the assessments as brief as possible, a subset of items from 3 standard mental health screening tools was used. Item-level depression, anxiety, and PTSD symptom data from a prior study of 232 VHA primary care patients (Carlson et al, unpublished data, 2021) and from the first 17 participants of this study were analyzed using multivariate regression to select subsets of items to assess these symptoms. Depression was assessed with 5 of the original 9 Patient Health Questionnaire-9 [24] items (anhedonia, feeling down, trouble sleeping, feeling like a failure, and trouble concentrating), resulting in a 5-item scale with high internal consistency (α=.80). Anxiety was assessed using 4 of the original 7 General Anxiety Disorder-7 items [25] (feeling nervous, worry, trouble relaxing, and restlessness), resulting in a 4-item scale with high internal consistency (α=.88). PTSD was assessed using 9 of the 17 original items from the Screen for Posttraumatic Stress Symptoms [26,27] (avoiding thoughts, avoiding situations, nightmares, feeling shaky, irritability, surroundings feeling unreal, remembering awful things, upset upon reminders, lost track of what is happening around me, flashbacks, and things seeming unreal), resulting in a 9-item scale with high internal consistency (α=.88). Scores on the briefer measures correlated between .96 and .97 with scores on the full measures in the 2 data sets. Problem-solving knowledge was assessed using 6 items related to the information included in the course (eg, multiple choice questions on how stress can affect problem-solving, good strategies for problem solving, and how to evaluate possible solutions). Problem-solving confidence was assessed using 3 items inquiring about agreement with statements regarding best ways to solve problems, feeling confident about solving future problems, and having strong problem-solving skills, all answered on a 4-point scale ranging from *I don’t agree* to *I agree a lot*. The Client Satisfaction Questionnaire comprised 8 items administered at the end of the study and assessed satisfaction with the services they received without differentiating among recruitment, assessment, and intervention services [28].

Qualitative interviews were conducted post treatment to assess participant reactions to MF with and without PS. Results related to which aspects of PS were most useful or least useful and perceptions of working with a peer versus a traditional mental health provider have been included in this report.

### The MF Course

MF is an instructionally sound, web-based self-help program [29]. It was built collaboratively by VHA and DoD as part of a coordinated public health initiative to address the unmet mental health needs of returning service members. To reduce any barriers to use, it is completely free, anonymous, and available to the public. Users do not have to register or provide any personal information to use the program. A version of the course is available on the web [29]. The MF web-based intervention was based on a 4-session problem-solving workshop that has shown efficacy in affecting key targets [30] and makes extensive use of video, animation, and interactive exercises to engage participants. Its design was informed by the principles of adult learning theory and closely follows best practices in instructional systems design [31]. It includes detailed explanations of the relationships among thoughts, behaviors, and moods. The program places a strong emphasis on completing problem-solving worksheets and other tasks between sessions and uses a variety of techniques to help users monitor and challenge unhelpful thoughts. MF is based on cognitive behavioral treatment principles; however, to reduce stigma, it is presented to veterans as an *educational and life coaching program* that teaches skills and tools to solve stressful problems and overcome obstacles. Videos show highly relatable, demographically diverse characters applying problem-solving principles to address problems that veterans commonly experience, such as financial difficulties and relationship distress. Table 1 includes the major components of each module. The modules are self-paced but typically take approximately 20 minutes each to complete.

**Table 1:** Moving Forward modules and peer sessions.

Moving Forward content	Peer sessions (fidelity elements)
—^a^	Session 1 Help the veteran choose a problem to focus on Ask the veteran to complete module 0
Module 0: What type of problem solver are you? Common problem-solving challenges, interactive stress game, introduction to problem solving Problem-solving attitudes and approaches—vignettes and explanations Optimistic versus pessimistic problem-solving attitudes Problem-solving approaches—thoughtful planner, quick-fixer, and avoider Problem-solver questionnaire and feedback—identifying strengths and weaknesses and getting personal insight into how one handles stressful situations	Session 2 Ask about module completion Discuss veteran’s problem-solving strengths and weaknesses Ask the veteran to complete modules 1 to 2
Module 1: Solve problems when your brain is overloaded How our brains get overloaded and our limited ability to multitask How to *externalize, simplify, and visualize* to minimize *brain overload* Module 2: Solve problems under stress Survey to measure stress level How stress affects your mind, body, and behavior (and problem solving) Stop and Slow Down steps toolkit with videos (eg, relaxation exercises) and a game (Rocket Commander) to demonstrate steps	Session 3 Ask about module completion Discuss the following: Strategies to try when the veteran experiences *brain overload* How stress affects problem solving Any relaxation strategies veteran tried Ask the veteran to complete module 3
Module 3: Solve problems step-by-step *Planful* problem solving to focus on the Think and Act steps of problem solving Interactive problem-solving worksheets—fillable exercise to define the problem, obstacles, and courses of action Videos on evaluating and selecting courses of action	Sessions 4 Ask about module completion Discuss the following: Implementation of the problem-solving action plan Overcoming barriers to the action plan Create a new problem-solving worksheet, if applicable Ask the veteran to keep working on their action plan and complete module 4
Module 4: Where to go from here Emphasizes practice and anticipating future problems; interactive game to illustrate this concept Encourages celebrating positive progress Encourages veterans to keep trying and offers vignette videos to demonstrate positive outcomes of using the program Offers examples of when and how to reach out for more help	Session 5 Discuss the following: Implementation of the problem-solving action plan Overcoming barriers to the action plan Review skills developed in the program Develop a plan to continue problem solving without peer support Connect to additional resources, as needed

^a^The participants began using Moving Forward after session 1 so no content is included in this cell.

### Peer Support

The peer sessions were designed to be approximately 20-minute phone calls. Over 8 weeks, 5 sessions were offered to participants, and the spacing between sessions was based on participant preference. The session content was guided by the PS guide, which was adapted from a previous study of PS for a web-based mental health program [32]. The goals of the peer sessions were to help the participants fully engage in the MF content and apply it to daily life. Peers aimed to engage participants in meaningful discussions on how to apply problem-solving skills and share their own experiences of overcoming life’s problems, as appropriate. Each session had essential fidelity elements, as detailed in Table 1. Peers used REDCap templates to document sessions and endorse the fidelity elements that were met. A total of 2 peers provided support for the study: both had experience working as peer specialists with veterans and completed study-specific training that included didactics, personally using MF, and receiving feedback on role-plays of sessions. Peers also participated in weekly group supervisions led by a clinical psychologist with peer supervision expertise.

### Analyses

Feasibility and acceptability metrics were described using means and frequencies. Feasibility metrics included the rate of participant enrollment per month and the rates of assessment completion at baseline and at the end of the study. Peer fidelity was described as the percentage of fidelity elements completed. User acceptability metrics included the percentage of participants who logged in to use MF, percentage of participants who had any peer sessions, number of peer sessions completed, and participant satisfaction. Satisfaction among participants who did and did not have access to MF was compared using a 2-tailed *t* test. Poisson regression with a Pearson adjustment to the SE was used to compare the number of log-in days (a count variable) between the SD and PS conditions. Multilevel modeling (MLM) via SAS Proc Mixed was used to compare changes in problem-solving knowledge and problem-solving confidence among participants who used and did not use MF. MLM was also used to estimate the associations between problem-solving confidence and depression, anxiety, and PTSD symptom outcomes. MLM was also used to compare changes in mental health symptoms among participants who engaged in peer sessions with those who did not. Analyses were adequately powered for the primary (to test the feasibility and user-perceived acceptability of the methods) and secondary (to examine engagement in the web-based MF course for those with and without PS) aims. Our planned sample size did not provide sufficient power to detect differences between conditions in mental health symptoms; therefore, group-by-time analyses were not conducted.

Qualitative data were analyzed using a rapid analysis approach [33,34]. Templated summaries of interview responses were created and then entered into a matrix, with a domain name for each survey question placed on the horizontal axis and participants listed on the vertical axis. A total of 2 authors (JW and KP) independently began data reduction on discrete copies of the matrices to develop concise summaries that focused and organized the data. The authors then met to compare their data reductions and create a final matrix that reflected their agreed-upon themes.

## Results

### Sample Characteristics

A total of 81 veterans recruited from social media posts were enrolled and randomized to study conditions (PS 27/81, 33%; SD 28/81, 35%; WL 26/81, 32%). Participant characteristics are shown in Table 2. Participants lived in 38 different states, and most enrollments (60/81, 71%) occurred outside of typical office hours within the veterans’ home time zone. The participants reported significant unmet mental health needs based on our definition in this study.

**Table 2:** Characteristics of the participants (N=81).

Variable		Participants
Age (years), mean (SD; range)		54 (9.4; 30-77)
**Gender, n (%)**		
	Male	48 (59)
**Race and ethnicity, n (%)**		
	White	60 (74)
	Black	8 (10)
	Latinx	8 (10)
	Mixed race	8 (10)
	Native American	8 (10)
	Asian or Pacific Islander	3 (4)
**Marital status, n (%)**		
	Single	7 (9)
	Married or partnered	36 (44)
	Separated or divorced	30 (37)
	Widowed	4 (5)
**Education, n (%)**		
	High school	11 (14)
	Some college or vocational school	49 (60)
	Bachelor’s degree or more	31 (26)
**Employment status, n (%)**		
	Employed	25 (31)
	Disabled	22 (27)
	Retired	16 (20)
	Unemployed	11 (14)
	Student or homemaker	4 (5)
**Service branch, n (%)**		
	Army	96 (51)
	Navy	42 (22)
	Air Force	32 (17)
	Marines	19 (10)
	Coast Guard	1 (1)
**Mental health, n (%)**		
	Endorsed all 5 symptoms of depression	35 (43)
	Endorsed ≥3 symptoms of depression	67 (83)
	Scored above clinical cutoff for depression on Patient Health Questionnaire-2 [35]	55 (68)
	Endorsed ≥3 anxiety symptoms	66 (81)
	Endorsed ≥6 posttraumatic stress disorder symptoms	53 (65)
	Endorsed problems with sleep	74 (91)

### Feasibility

Figure 1 shows the rates of participant recruitment, screening, enrollment, assessment, and intervention allocation. The web-based recruitment method yielded 13.5 participants per month over a 6-month period. Only 24% (19/81) of participants completed the follow-up assessment after receiving automated emails. The end of the study assessments were ultimately completed by 46% (37/81) of the participants following research assistant phone calls. Assessment completion rates were 30% (8/27) for PS, 39% (11/28) for SD, and 69% (18/26) for WL conditions. Baseline characteristics (eg, gender, race, age, and depression, anxiety, or PTSD symptoms) did not significantly differ among participants who completed the follow-up assessment and those who did not across the entire sample or within randomized conditions.

### Peer Sessions and Fidelity

Just over half (15/27, 56%) of the participants assigned to receive PS participated in sessions with peers. Of the 15 participants, 4 (15%) received 1 session, 7 (26%) received 2 to 4 sessions, and 4 (15%) received all 5 peer sessions. Moreover, 1 peer engaged and provided support sessions to 65% (12/18) of those assigned to work with him and had a mean of 2.9 (SD 1.6) sessions with the participants he did have sessions with, whereas a second peer provided sessions to 42% (3/7) of those assigned and had a mean of 2.2 (SD 1.4) sessions with those he did have sessions with. Peer sessions had 100% fidelity to the Peer Guide, indicating that the peer training methods combined with the REDCap documentation system were highly feasible for peers to complete.

### User Acceptability

Most participants in the SD and PS conditions logged into the course at least once (19/27, 70% and 20/28, 71% of participants, respectively). Participant satisfaction was favorable in both the SD and PS conditions. Satisfaction was significantly higher in the SD and PS conditions compared with the WL condition (t_35_=−2.54; *P*=.03) and did not differ between the SD and PS conditions. Specifically, 95% (18/19) of participants in the SD and PS conditions who completed the satisfaction measure rated the overall quality as good to excellent and said that they received the kind of services they wanted. Similarly, 90% (17/19) said that they would recommend the program to a friend.

Of the 8 PS participants who completed the follow-up interview, 4 (50%) agreed to the interviews, and 3 (38%) completed the interviews, all of whom reported highly positive feedback. Regarding their overall experience with the peer, participants noted that the peer they worked with was *extremely helpful*, encouraged them to continue in the course, and helped them feel like they were *not alone* in their struggles. The most helpful aspects of PS included having the peers clarify course content and suggest new ideas for problem solving when the participants felt stuck. A veteran noted, “having interaction with the website is one thing, but having someone to talk to and getting feedback is really helpful and supportive.” Participants commented that their peer’s veteran status made them feel like they had more in common with the peer and that the peer could understand their struggles more. A total of 2 suggestions for improvements were noted: 1 participant said that the 20-minute phone sessions felt “a bit rushed” and that having a picture of the peer would be helpful as they only spoke by phone.

### Impact of PS on Course Use and Mental Health Symptoms

Table 3 shows the results of the analyses of course use. Course use was significantly greater for PS participants than for SD participants. The effect of PS was more pronounced among participants who engaged in at least one peer session; participants who had one or more PS sessions logged in on significantly more days than participants who had access to MF but had no PS sessions. Participants who had at least one PS session had larger decreases in depression than those who did not have a PS session. Participants who engaged in peer sessions did not have significantly different changes in anxiety symptoms compared with those who did not have peer sessions.

**Table 3:** Impact of peer support on course use and mental health symptoms (N=81).

Outcome			Values, n (%)		Values, mean (SE)		B (SE)		*P* value
**Peer support**									
	Course use (days logged in)	27 (33)		2.96 (0.22)		−0.89 (0.41)		.03	
**Self**−**directed**									
	Course use (days logged in)	28 (35)		1.21 (0.67)		−0.89 (0.41)		.03	
**Any peer sessions**									
	Course use (days logged in)	15 (19)		4.87 (0.18)		1.56 (0.31)		.001	
	Depression symptoms change	15 (19)		−4.3 (1.93)		−6.73 (2.04)		.002	
	Anxiety symptoms change	15 (19)		−1.8 (2.17)		−3.88 (2.30)		.10	
**No peer sessions**									
	Course use (days logged in)	42 (52)		1.02 (0.25)		1.56 (0.31)		.001	
	Depression symptoms change	42 (52)		2.4 (0.68)		−6.73 (2.04)		.002	
	Anxiety symptoms change	42 (52)		2.1 (0.77)		−3.88 (2.30)		.10	

### Associations of Problem-Solving Confidence With MF Use and Mental Health Symptoms

Participants who used MF at least once (including those in the PS and MF conditions) had larger increases in problem-solving confidence compared with participants who did not use MF (2.4 vs −0.72; B=−1.72, SE 0.64; *P*=.01). Problem-solving knowledge did not differ by whether participants used MF (B=−0.35, SE 0.51; *P=*.31). In addition, a 1-point increase in problem-solving confidence was associated with a 1.12-point decrease in depression (B=−1.12, SE 0.20; *P*=.001), a 0.82-point decrease in anxiety (B=−0.82, SE 0.20; *P*=.001), and a 1.51-point decrease in PTSD severity (B=−1.51, SE 0.45; *P*=.001).

## Discussion

### Principal Findings

This paper reports on the feasibility and user-perceived acceptability of innovative and automated methods for recruiting, enrolling, assessing, and delivering a web-based problem-solving intervention (MF) to veterans with unmet mental health needs. The results also demonstrate the impact of PS on MF use and describe the relationships between multiple potential mechanisms and outcomes of web-based problem-solving training to inform future research.

The methods for social media recruitment were successful in recruiting a sample of veterans in the community with unmet mental health needs who were not engaged in mental health care in the previous 6 months. The sample was diverse in geographic location, age, gender, race, and ethnicity and resulted in the recruitment of many women and younger veterans who can be challenging to recruit with in-person recruitment methods in VHA. In addition, most participants enrolled in the evening or on a weekend day, indicating that they preferred to use the service outside of regular business hours.

Our automated systems for screening, enrollment, administration of the initial assessment, randomization, and condition assignment created a seamless, brief, and easy user experience that led to the enrollment of our targeted number of eligible participants. The automated collection of course use data gathered objective information about whether the course was accessed and how many days participants logged in to the course. The results indicated that the automated enrollment and assessment methods led to acceptable levels of eHealth engagement [36]. Our study found a similar or lower percentage of nonusers (16/55, 29%) compared with other eHealth studies, which found that 25% to 58% of the participants never used the intervention [37]. Clinical programs also often suffer from less-than-ideal engagement. For example, a report of a program found that less than half of primary care patients who screened positive for depression or PTSD and were offered mental health care received any sessions [4].

Given the widespread challenges of engaging individuals with mental health needs to use eHealth tools and mental health services more generally, our finding that PS was associated with increased MF use is significant. These results are supported by our qualitative findings, where participants described their peer relationships as encouraging and helpful in moving them through barriers in their own understanding of MF content or their ability to apply it to their current problems.

Our finding that 55% (15/27) of participants offered PS chose not to do phone sessions indicates that not all eHealth users want or need PS. It is possible that the delivery of PS by phone hampered uptake and that some users would have preferred messaging or video formats. An option for peer messaging or video chats may be feasible going forward, given the increase in remote delivery of services during the COVID-19 pandemic [7]. Satisfaction was also high in the SD MF condition, further indicating that many users did not see the need for additional support in using the modules. Although satisfaction results may be influenced by ceiling effects on the Client Satisfaction Questionnaire, taken together, these results signal that alternative study designs and stepped-care interventions may be more appropriate for studying the added benefits of PS to eHealth. For instance, a design that allows users to opt for PS if they want or need it or step up to additional support if they are not engaging in SD use could reveal how often users want and need additional support and if this additional support increases eHealth use among participants who cannot engage on their own.

The streamlined user experience created by the automated enrollment and assessment systems may have resulted in some veterans being enrolled and randomized who were not motivated to engage in follow-up assessments, as evidenced by our assessment attrition rate of 54%. High attrition from eHealth studies is common and well-described in the literature [16]. Efforts to boost follow-up assessments can include reducing participant burden (ie, number of questions) in assessments or adding interactions with research staff at enrollment via email, text, or phone to confirm that the participant truly intended to fully participate. Interaction with research staff can also build a participant’s commitment to a study. A recent meta-analysis found that mobile health studies that enrolled participants on the web have much higher attrition (43%) than those that use in-person (11%) and phone (18%) enrollment [37]. Although adding contact with research staff is likely to decrease attrition, therefore increasing the internal validity of a study, it may also make the user experience more complex and less reflective of real-world eHealth use, decreasing the external validity of a study; that is, veterans who could benefit most from web-based programs may not meet a higher bar for motivation. Furthermore, adding contact with staff will increase study costs and decrease the number of people who can be reached. Ultimately, researchers may need to anticipate high assessment attrition in eHealth studies that use automated enrollment procedures and increase enrollment with the goal of still having an adequate sample size at the end of the study.

Our findings were consistent with the results of a meta-analysis that found higher assessment attrition in active conditions compared with WL controls and concluded that this might be because participants in active conditions have full access to the eHealth intervention and therefore are less motivated to complete assessments than those in the WL control who need to complete assessments before gaining access [37]. An additional explanation is that the active conditions (especially PS in our study) required more participant effort and time than the WL control; therefore, WL participants may have been more willing to spend additional time on the assessments.

Confidence in problem-solving skills appears to be an important correlate of increased course use and changes in mental health symptoms. Confidence and the closely related construct of self-efficacy are common mechanisms of action in many behavioral interventions, including eHealth programs [14,38]. Future research should examine problem-solving confidence or mental health self-efficacy as a mediator between MF use and mental health outcomes and consider optimizing components of the intervention that increase confidence, such as peer encouragement to practice problem solving, as more practice is likely to lead to more mastery and confidence.

Engagement in PS was associated with decreases in depression. This is consistent with emerging research showing the mental health benefits of PS [39]. There are many possible mechanisms that may explain the relationship between PS and mental health outcomes that are well-described in the supportive accountability model, including that being accountable to a supportive and knowledgeable individual increases engagement with eHealth or self-help tools [17]. Peers add an additional component of shared experience that can enhance the strength and benefits of the relationship.

This study has several strengths and limitations. Automated methods were successfully used to recruit, screen for inclusion, obtain consent, and enroll veterans with unmet mental health needs to a study at rates comparable with other eHealth studies and referral in primary care. Baseline and course use data were also successfully collected using automated methods. Web-based recruitment resulted in a sample that was diverse in age, gender, service branch, service era, and geographic location. The study successfully randomized some participants to receive PS for the course and tracked remotely provided PS contacts and sessions. A major weakness of the study was the low rate of completion of the study end assessments. Automated requests to complete the assessments were not sufficiently effective to achieve adequate completion rates, and staff phone calls to encourage completion were inconsistent. To increase the study end measure completion in future studies, staff efforts to reach participants could be increased, and data might be collected by study staff by phone. Given the relatively low completion rate for the study end measures, it is possible that more satisfied participants completed measures at higher rates than less satisfied participants, possibly leading to a positive bias in both quantitative and qualitative satisfaction results. An additional limitation was that the success in engaging participants was lower for one of the peer specialists. Future studies on the impact of PS on eHealth use should consider increasing the number of PS providers and assessing factors such as participants’ perceptions of therapeutic alliance with peers.

### Conclusions

In conclusion, automated processes for recruiting, enrolling, screening, assessing, and providing a cognitive behavioral eHealth intervention are feasible and acceptable overall; however, additional efforts are necessary to achieve adequate study end assessment completion rates. The finding that delivering PS for MF by phone with high fidelity was feasible and the increased use of the course indicates that PS may boost engagement with web-based courses. Innovative designs such as SMARTs (Sequential Multiple Assignment Randomization Trials) may clarify for whom PS is most helpful, when it is most helpful, and what outcomes are likely to improve. Finally, the associations of problem-solving confidence with both course use and improved mental health outcomes indicate that problem-solving confidence shows promise as a potential mechanism of action in future eHealth research.

## Abbreviations

DoD: Department of Defense
MF: Moving Forward
MLM: multilevel modeling
PS: peer support
PTSD: posttraumatic stress disorder
REDCap: Research Electronic Data Capture
SD: self-directed
SMART: Sequential Multiple Assignment Randomization Trial
VHA: Veterans Health Administration
WL: waitlist
